# Construction of an early warning model for venous thromboembolism risk in patients with severe cerebral hemorrhage based on ultrasound spontaneous imaging

**DOI:** 10.3389/fneur.2025.1562963

**Published:** 2025-06-04

**Authors:** Bei Ma, Chen Chen, Qin Wang, Xi Chen

**Affiliations:** Department of Critical Care Medicine, Liangjiang Hospital Affiliated to Chongqing Medical University, Chongqing, China

**Keywords:** ultrasound spontaneous echo contrast, spontaneous cerebral hemorrhage, venous thromboembolism, warning model, albumin

## Abstract

**Objective:**

To investigate the role of ultrasound spontaneous echo contrast (SEC) in venous thromboembolism (VTE) in patients with severe spontaneous cerebral hemorrhage (ICH) and to construct a clinical prediction model.

**Methods:**

A total of 69 critically ill ICH patients admitted to the Department of Critical Care Medicine of Liangjiang Hospital of Chongqing Medical University between January 2022 and March 2024 were included in the study. Datas were collected prospectively, including general information, test data, clinical outcomes, and lower limb vascular ultrasound images within 48 h of admission. The statistical analysis was conducted using SPSS 22.0, and the model was constructed using binary logistic regression analysis. The efficacy of the model was assessed using subject operating (ROC) curves and the Hosmer-Lemeshow goodness-of-fit test.

**Results:**

The SEC, Albumin and age were identified as independent risk factors for thrombosis in patients with severe ICH. The joint prediction model, constructed based on the indicators, is given by the following equation: Logit(P) = 0.477–0.216 * Albumin + 1.43 * SEC + 0.044 * age. The model demonstrated consistent predictive performance, exhibiting good discrimination (AUC = 0.900) and calibration (Hosmer-Lemeshow χ2 = 5.231, *p* = 0.733 > 0.05).

**Conclusion:**

The ICH-VTE early warning model constructed on the basis of SEC, Albumin and age performs well and helps clinicians to dynamically assess the risk of VTE to determine the timing of anticoagulation, which provides therapeutic ideas to reduce the incidence of VTE and improve the clinical outcome of ICH.

## Introduction

Venous thromboembolism (VTE), which encompasses both pulmonary embolism (PE) and deep venous thrombosis (DVT), represents a prevalent and grave complication among patients with intracerebral hemorrhage (ICH) (1). Prophylactic anticoagulation represents an efficacious strategy for the reduction of VTE. However, concerns pertaining to the potential risk of secondary cerebral hemorrhage following anticoagulation have led to the frequent postponement or even absence of anticoagulation in critically ill ICH patients. This represents a significant challenge, both domestically and internationally (2). Current thromboembolic risk assessment tools, including the Padua Prediction Score, the Improved Risk Score for VTE in Stroke, the Caprini Score and the Wells Score, have significant limitations when applied to ICH patients. Many of these scores were developed for mixed stroke populations (ischemic and hemorrhagic) or surgical patients, and have not been validated in pure ICH cohorts. Furthermore, these scoring systems predominantly comprise static models that fail to account for the dynamic nature of VTE risk in ICH patients. Lastly, a reliable scoring system for predicting severe ICH-VTE risk to guide anticoagulation is currently lacking, underscoring the importance of exploring early warning indicators that can identify VTE risk in severe ICH (3).

Spontaneous echo contrast (SEC) represents a phenomenon of overlapping and accumulation of red blood cells, which is caused by the reduced shear force of blood flow when blood flow is slowed down or obstructed. This results in the formation of cloudy echoes on ultrasound. The formation of SEC in the left atrium is regarded as an independent risk factor for the development of thrombosis and stroke (4). A recent study has revealed that the prevalence of venous thromboembolism (VTE) is significantly higher in patients with severe neocoronary pneumonia and positive SEC results (5). The observed difference was found to be statistically significant, indicating that SEC may serve as a potential precursor to VTE. Consequently, the study recommends the implementation of active anticoagulation therapy. The present study was designed to further elucidate the early warning role of SEC severity in the development of VTE after ICH and to construct a clinical prediction model to guide anticoagulation therapy after cerebral hemorrhage.

## Materials and methods

### Study design

**Figure 1:**
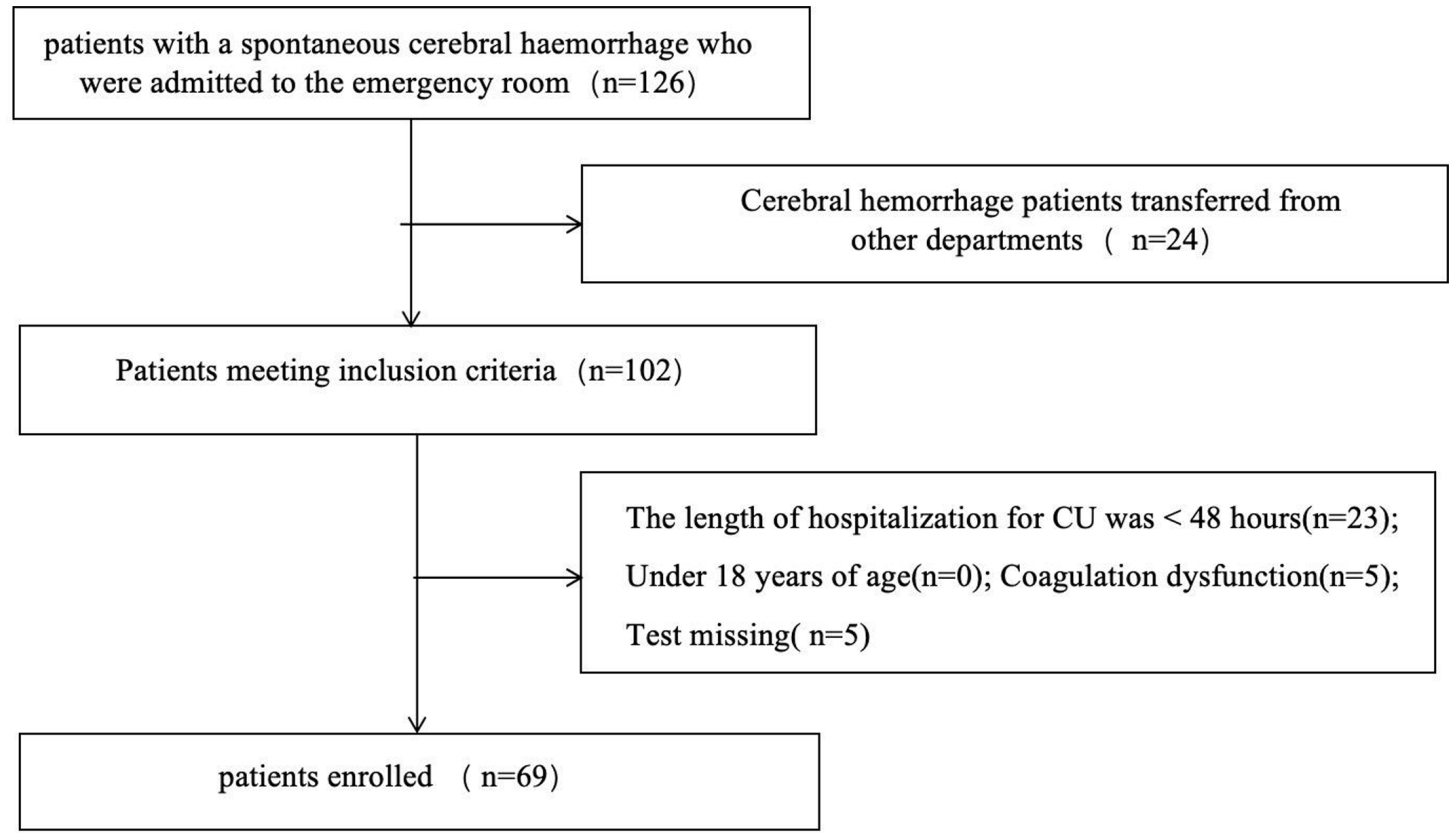
Flowchart of the study.

The study population comprised prospectively collected patients with cerebral hemorrhage admitted to the Department of Intensive Care Medicine of Chongqing Liangjiang New District People’s Hospital from January 2023 to March 2024. The study was approved by the Ethics Committee of Chongqing Liangjiang New District People’s Hospital. Severe intracerebral hemorrhage (ICH) refers to cases requiring ICU admission. The criteria for ICU admission are as follows (meeting any one qualifies): ① Depressed consciousness: Glasgow Coma Scale (GCS) score ≤12 (or ≤8 with requirement for endotracheal intubation/mechanical ventilation). ② Large hematoma volume: Supratentorial hemorrhage > 30 mL, or Infratentorial hemorrhage > 10 mL, or Radiological mass effect (e.g., midline shift ≥ 5 mm). ③ Brainstem hemorrhage: Direct compromise of vital brainstem functions. ④ Hemodynamic instability: Need for continuous vasoactive drugs to maintain blood pressure, or Severe hypertension (e.g., systolic blood pressure > 220 mmHg) requiring intensive management. ⑤ Respiratory failure: Impaired airway protection (e.g., diminished cough reflex), or Hypoxemia (PaO_₂_ < 60 mmHg) with/without mechanical ventilation. The exclusion criteria were: (1) the presence of a VTE on admission; (2) a length of stay in the intensive care unit of less than 48 h; (3) the presence of severe coagulation dysfunction, as evidenced by an INR level greater than 2.0 or a platelet count of less than 50 × 10^3^/μL; and (4) inability to complete the requisite tests and examinations. In total, 69 patients were included and divided into two groups according to ultrasound diagnostic criteria: one group with VTE and one without (Figure 1).

### Data collection

The following data were collected: general information, comorbidities, Acute Physiology and Chronic Health Evaluation (APACHE II) score, Glasgow Coma Scale (GCS) score, venous thromboembolism (VTE) score, and laboratory data. Lower limb vascular ultrasound images were obtained at the bedside within 48 h of admission. The survival outcomes of the patients were documented and the result of the mortality rate was defined as the total mortality rate within 90 days. The data should be entered and organized using Excel software.

The following points are of particular significance: Lower limb vascular ultrasound is conducted by a critical care medicine physician who is qualified in critical care ultrasound, utilizing a GE VENUE ultrasound machine (General Electric, United States), equipped with a high-frequency (>7 MHz) line array probe for the purpose of performing B-mode two-dimensional imaging of the lower limb veins (bilateral femoral and popliteal veins). In order to obtain B-mode images with clear contours of the vascular lumen and no obvious adjacent tissue artifacts, the time gain compensation (TGC), depth and gain were adjusted. A bespoke grading system was employed for the visual quantification of SEC (6) (Supplementary Figure 1). The images were scored by two other experienced physicians, with the scoring process blinded to the clinical data.

### Statistical analysis

The statistical analysis was conducted using SPSS 22.0 and MedCalc software. Data that exhibited a normal distribution were expressed as a t-test for comparison between groups. In the case of data that did not meet the criteria for normal distribution, the median (Q1, Q3) was used for expression purposes. The Kruskal-Wallis H rank sum test was employed for the purpose of comparison between groups. In the case of count data, the frequencies (rates) were expressed, and the chi-square test or Fisher’s exact test was employed. A multifactor logistic regression analysis (*α* in = 0.05, α out = 0.10, test level α = 0.05) was employed to screen the identified variables and construct the model. The performance of the model was evaluated using receiver operating characteristic (ROC) curves. Z-tests were employed to ascertain the statistical significance of the differences in the area under each ROC curve. Additionally, the Hosmer-Lemeshow goodness-of-fit test was utilized to assess the calibration ability of the models. A *p*-value of less than 0.05 was considered statistically significant.

## Result

### Description of demographic characteristics

A total of 69 cases were included in this study, comprising 45 cases in the non-VTE group and 24 cases in the VTE group. A total of 24 patients with cerebral hemorrhage were identified as having VTE, including 23 with DVT and 2 with PE, and 1 with both DVT and PE. The median time from admission to VTE was 5.5 days (IQR 4–8 days), with the earliest time of VTE formation being 2 days after intracerebral hemorrhage. Following the diagnosis of VTE and the initiation of therapeutic anticoagulation, three patients experienced a bleeding event. These comprised one patient with airway hemorrhage, two patients with fecal occult blood, and no significant increase in intracerebral hematoma. The VTE group exhibited a higher mean age and longer mean total hospital stay compared to the non-VTE group, with these differences being statistically significant (Table 1). Additionally, statistically significant differences were observed in the levels of SEC, CRP, D-dimer, Fib, Albumin, Cr and uric acid between the two groups (Table 2).

**Table 1:** Population description and comparison.

	non-VTE (n = 45)	VTE (n = 24)	Statistical value *t/Z/χ2*	*p*-value
Age	55.87 ± 17.37	67.58 ± 12.37	2.928	**0.0047**
Male	31 (68.89%)	14 (58.33%)	0.8768	0.3806
Hypertension	22 (48.89%)	13 (54.17%)	0.4177	0.6762
Diabetes	1 (2.22%)	2 (8.33%)	1.794	0.0728
COPD	0	1 (4.17%)	1.379	0.1678
Stroke	4 (8.89%)	2 (8.33%)	0.078	0.9378
Coronary heart disease	1 (2.22%)	1 (4.17%)	0.459	0.6466
Chronic renal failure	2 (4.44%)	2 (8.33%)	0.658	0.5103
Tumor	1 (2.22%)	0	0.736	0.4619
Smoking	20 (44.44%)	9 (36%)	0.557	0.5778
Apache II	22.86 ± 6.16	23.38 ± 5.32	0.343	0.7328
R value	37.28 (20.33,56.76)	42.79 (28.25,64.405)	0.918	0.359
GCS	7 (5,9)	6 (5,9.75)	0.573	0.567
Blood transfusions	11 (24.44%)	8 (3.33%)	0.787	0.431
ICU stay (Day)	8.55 (5.41,12.45)	8.46 (5.67,13.94)	0.454	0.65
Total hospital stay (Day)	19 (8.5,47)	43 (15.25,95.75)	2.678	**0.007**
Hospital costs (¥)	139,203 (81,196,225,576)	18,826 (80,299,287,262)	1.222	0.222
Death	14 (31.11%)	5 (20.83%)	0.9103	0.3627

Bold *p*-values in the text indicate statistically significant results with *p* < 0.05, suggesting strong evidence against the null hypothesis.

**Table 2:** Clinicopathological characteristics of 69 patients.

	non-VTE (n = 45)	VTE (n = 24)	Statistical value *t/Z*	*p*-value
SEC	1.36 ± 1.18	2.5 ± 1.14	3.4	**0.0012**
White blood cell count (10^9^/L)	12.37 ± 4.34	12.71 ± 3.71	0.8487	0.3991
Increased neutrophil percentage (%)	87.27 ± 7.86	84.89 ± 5.77	0.8065	0.4228
CRP (mg/L)	37.97 ± 58.07	82.47 ± 73.62	2.805	**0.0066**
PCT (ng/ml)	0.89 ± 1.42	0.65 ± 1.19	0.5961	0.5542
PLT (10^9^/L)	182.4 ± 57.31	211.0 ± 158.1	1.090	0.2796
PT(S)	13.6 (12.33,13.80)	12.4 (11.3,14)	1.399	0.162
APTT(S)	31.25 (27.5 ± 33.03)	29 (25.9,34.20)	1.834	**0.067**
PTA (%)	89.88 ± 14.39	88.70 ± 10.94	0.007	0.9943
D-Dimer (ng/mL)	1.2 (0.5,3.63)	2.96 (1.89,3.91)	2.721	**0.007**
Fib (g/L)	2.69 (2.23,3.78)	3.88 (3.26,5.22)	2.848	**0.004**
BNP (pg/mL)	89 (37,418.5)	85 (65.1,163)	0.042	0.966
TnI (ug/L)	0.13 ± 0.47	0.041 ± 0.085	0.553	0.580
Myo (ug/L)	235.26 (71.76,872.88)	87.78 (35.71,206,31)	1.523	0.128
ALT (U/L)	18 (13,22)	25 (17,44)	0.996	0.319
Albumin (g/L)	37.77 ± 13.62	31.2 ± 4.35	2.278	**0.026**
Cr (umol/L)	77 (54.3,109)	59 (52.2,69.5)	1.758	0.079
Uric acid (mg/dL)	289.50 (188,399)	210 (143,264)	1.951	0.051

Bold *p*-values in the text indicate statistically significant results with *p* < 0.05, suggesting strong evidence against the null hypothesis.

### Logistic regression screening variables

Univariate logistic regression analysis indicated that age, spontaneous echo contrast (SEC), C-reactive protein (CRP), fibrinogen, Albuminumin, creatinine and uric acid had a statistically significant impact on the incidence of venous thromboembolism (VTE), with the exception of D-dimer. Multifactorial regression analysis demonstrated that age, SEC and Albuminumin were independent risk factors for VTE (Table 3).

**Table 3:** Logistic regression analysis.

Variables	Univariate analysis					Multivariate analysis				
	β	S. E	Wold	*p*	OR (95%CI)	β	S. E	Wold	*p*	OR (95%CI)
Age	0.05	0.02	7.13	0.008	1.05 (1.01~1.09)	0.04	0.02	3.57	0.059	1.05 (1.00~1.10)
SEC	1.17	0.30	10.14	<0.001	3.21 (1.77~5.82)	1.43	0.43	11.02	0.001	4.18 (1.80~9.72)
CRP	0.01	0.00	6.37	0.012	1.01 (1.01~1.02)					
D-Dimer	0.16	0.09	2.931	0.087	1.17 (0.98~1.40)					
Fib	0.37	0.15	6.352	0.012	1.45 (1.09~1.93)					
Albumin	−0.17	0.06	0.054	0.003	0.85 (0.76~0.94)	−0.22	0.08	7.369	0.007	0.81 (0.69–0.94)
Cr	−0.02	0.01	3.928	0.003	0.98 (0.96~0.99)					
Uric acid	−0.01	0.002	4.543	0.033	0.99 (0.99~0.99)					
Constant						0.477	2.70	0.031	0.860	

### Building a prediction model and evaluating its performance

**Figure 2:**
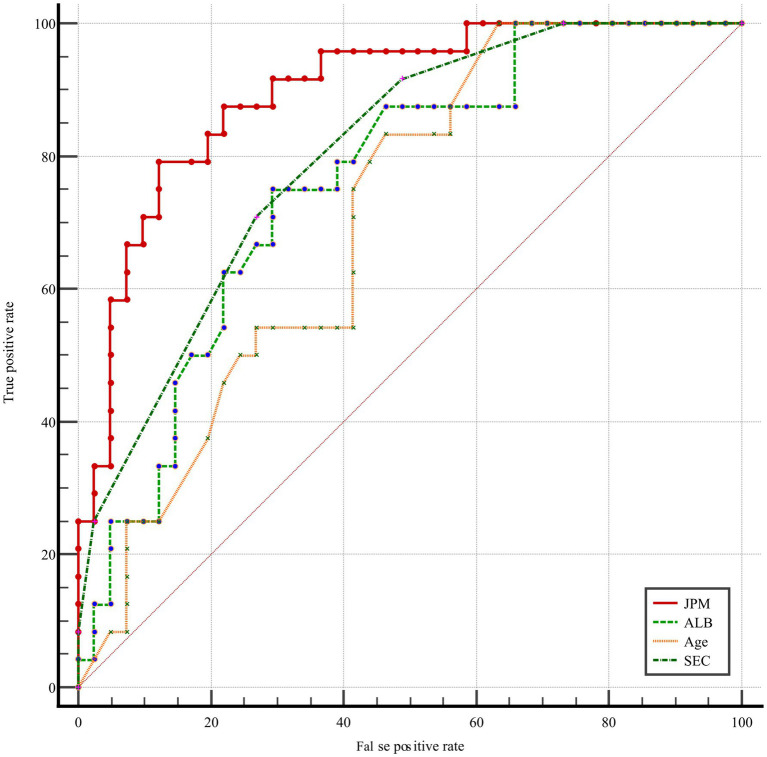
Receiver operating characteristic (ROC) curve.

**Figure 3:**
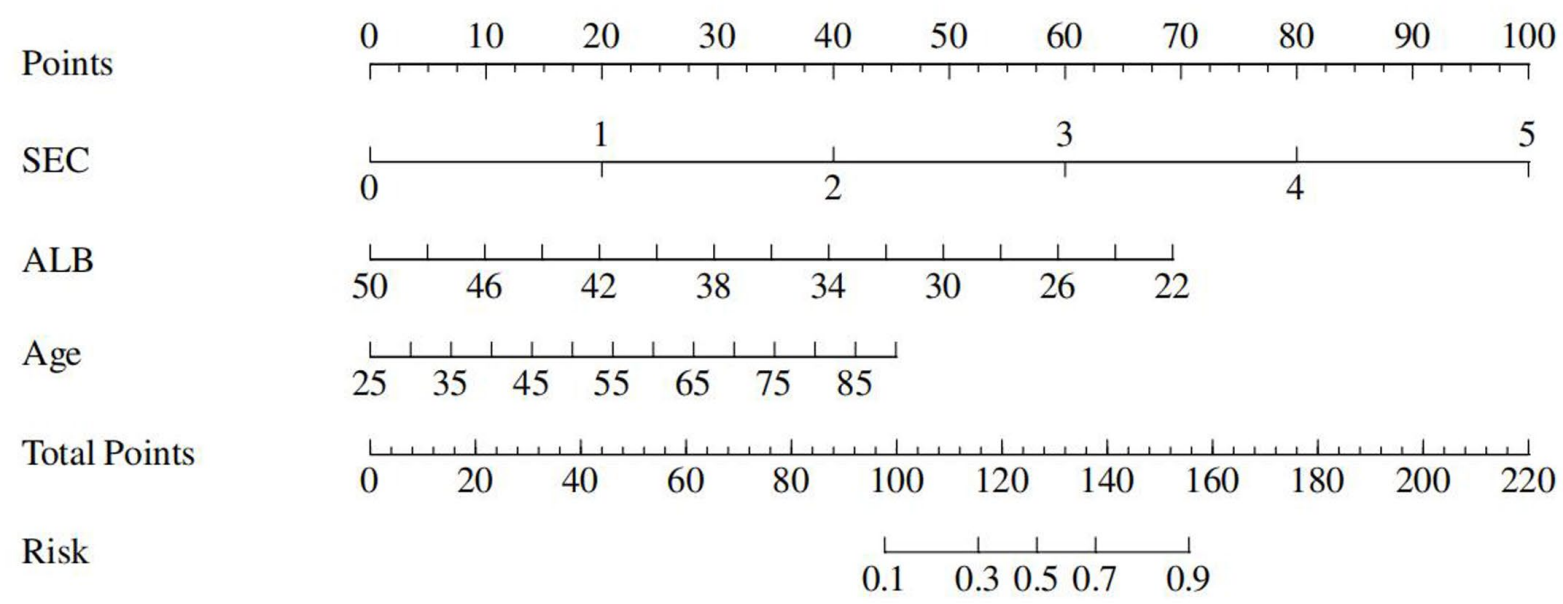
Nomogram.

A joint prediction model was constructed based on the *β* coefficient of logistic regression, which yielded the following equation: Logit(P) = 0.477 – 0.216 Albumin + 1.43 SEC + 0.044 * age. The ROC curves revealed that the joint prediction model (JPM) exhibited the greatest area under the curve (AUC = 0.900 > 0.817*0.699*0.735) (Figure 2), with a sensitivity of 87.5% and a specificity of 88.22% (Table 4). A comparison of the AUC of each ROC curve revealed a statistically significant difference in the area under the curve of the joint prediction model in comparison to the other groups (Supplementary Table 1). The Hosmer-Lemeshow χ^2^ statistic was 5.231, with a *p*-value of 0.733, which was greater than 0.05. This suggests that the discrepancy between the model-predicted values and the actual observed values was not statistically significant, indicating that the model had a good calibration. The results for the model’s independent variables are presented in Nomogram, as shown in Figure 3.

**Table 4:** Parameters associated with the receiver operating characteristic (ROC) curve.

Variables	*p*	AUC (95% CI)	Youden index	Cut-off point	Sensitivity (%)	Specificity (%)
SEC	<0.0001	0.817 (0.706~0.900)	0.472	≥2	91.67	55.56
Age	0.0014	0.699 (0.576~0.803)	0.367	>55	83.33	53.33
Albumin	0.003	0.735 (0.615~0.834)	0.417	≤33	75.00	66.67
	**<0.0001**	**0.900 (0.804~0.959)**	**0.697**	**>0.35**	**87.50**	**88.22**

Bold *p*-values in the text indicate statistically significant results with *p* < 0.05, suggesting strong evidence against the null hypothesis.

### Evaluate the performance of the model with subgroup analyses

To demonstrate the universality of the model, we add subgroup analyses to stratify risk and model performance by age and severity. We performed additional analyses stratifying patients into two age groups (≤55 vs. > 55 years) and by GCS score (≤8 vs. > 8) (Supplementary Table 2). The model consistently showed robust discrimination across subgroups (AUC = 0.988 for age ≤ 55 vs. 0.835 for age > 55; AUC = 0.891 for GCS ≤ 8 vs. 0.912 for GCS 9–12). These results have been included in the revised manuscript to demonstrate the generalizability of the model.

### Analyzing SEC risk factors with ordered logistic regression analysis of SEC risk factors

The results of the ordered logistic regression analysis indicated that CRP and Fib were independent risk factors for the formation and grading of SEC (Table 5).

**Table 5:** Ordered logistic regression analysis of SEC risk factors.

Variable	Regression Coefficient (B)	Standard Error (S. E.)	Wold statistic	*p*-value	95% CI
Albumin	−0.056	0.036	2.366	0.124	−0.127–0.015
Fib	0.323	0.142	5.164	**0.023**	0.044–0.602
D-dimer	0.160	0.100	2.596	0.107	−0.035–0.356
CRP	0.009	0.005	4.169	**0.041**	0.000–0.018
Age	0.023	0.015	2.332	0.127	−0.006–0.052

Bold *p*-values in the text indicate statistically significant results with *p* < 0.05, suggesting strong evidence against the null hypothesis.

## Discussion

The high incidence of venous thromboembolism (VTE) after intracerebral hemorrhage (ICH) results in adverse clinical outcomes (7). The lack of adherence to anticoagulation therapy is a common problem, and the early identification of patients at high risk of VTE for prophylactic anticoagulation is crucial for effective treatment. The authors analyzed data from severe ICH cases and identified SEC, age and Albumin as independent risk factors for VTE formation in severe ICH patients. The prediction model constructed by combining these three factors demonstrated good predictive value for severe ICH-VTE occurrence, suggesting potential as an early warning indicator of VTE and a guide for prophylactic anticoagulation treatment in severe ICH.

VTE is a prevalent and potentially fatal complication following a cerebral hemorrhage. The use of VTE prophylaxis may prove an effective method of enhancing clinical outcomes in individuals with intracerebral hemorrhage (ICH). At present, both domestic and international guidelines recommend the administration of prophylactic anticoagulant therapy for a period of 1–4 days following the onset of ICH, and only after the cessation of active bleeding (8). The difficulty in assessing the benefit of anticoagulation and the risk of bleeding, coupled with uncertainty about the optimal timing of anticoagulation (9), has resulted in prophylactic anticoagulation being administered to as few as 2% of ICH patients in clinical practice (2). The current versions of the Caprini and Padua scores lack sufficient specificity with regard to stroke, particularly in the case of severe stroke patients (10). The development of a risk model for severe ICH-VTE and the identification of high-risk patients for stratified management may prove to be important tools in the reduction of the incidence of ICH-VTE.

The risk factors currently most extensively studied in the context of ICH-VTE include advanced age, limb paralysis, postoperative intracranial hemorrhage, prolonged intubation, GCS score on admission, modified Rankin Scale (mRS) on discharge, length of hospital stay, and the highest levels of neutrophils, D-dimer, and Albuminumin (11, 12). Of these, age is a commonly used predictor of ICH-VTE (13), with approximately 70% of VTE occurring in people aged 60 years or older and the incidence increasing with age (14, 15). The relatively low age threshold for the risk of severe ICH-VTE (≥55 years) in the present study may be explained by the gradual tendency toward a younger population with cerebral hemorrhage. A low Albuminumin level (≤33) was identified as an independent risk factor for severe ICH-VTE in the present study, with a 24% increase in the risk of VTE for each unit of reduction. This finding is consistent with the results of previous studies in this field (16). The mechanism by which reduced Albuminumin increases the risk of VTE is more complex and may promote microvascular thrombosis by a number of different mechanisms. These include a decrease in colloid osmotic pressure, an increase in blood viscosity, an imbalance in the coagulation and anticoagulation systems, an interaction with inflammation to affect the integrity of the endothelial glycocalyx, and a promotion of endothelial damage (17, 18). It can therefore be proposed that hypoAlbuminuminaemia be included in the assessment of risk factors for VTE, as an indicator of an elevated risk of VTE at an early stage.

SEC represents a noteworthy phenomenon whereby the blood flow within the lumen manifests as a gradual procession of turbid, hyperechoic signals discernible on two-dimensional greyscale ultrasound. It is most frequently observed in the heart, internal jugular veins, and deep and superficial veins of the lower extremities. Prior investigations into SEC have concentrated on the assessment of thrombotic risk subsequent to cardiac disease and interventional procedures (19). The SEC is regarded as a significant predictor of both intra-atrial thrombosis and stroke (20). It has recently been demonstrated by foreign scholars that the formation of lower limb angiographic SECs in patients with severe cases of the novel coronavirus disease (2019-nCoV) may be a precursor to the development of venous thromboembolism (VTE). Consequently, they have recommended the implementation of an aggressive anticoagulation therapy (5, 21). In the present study, the severity of SEC was visually graded on a scale of 0 to 4 using semi-quantitative analyses. Furthermore, the formation of SEC and its effect on severe ICH-VTE were investigated (6). The results demonstrated that fib and CRP were independent risk factors for SEC formation, indicating that SEC may be responsive to hypercoagulable and inflammatory states within the blood. SEC was identified as an independent risk factor for ICH-VTE, and following adjustment for other variables, the risk of VTE was increased by 4.1. The odds of developing VTE increased by 78-fold for every 1-unit increase in SEC (95% CI = 1.796–9.718). Furthermore, the threshold for predicting VTE was level 2, exhibiting high sensitivity (91.67%) but low specificity (55.56%). In order to optimize the prediction model, a joint prediction model based on SEC, age and Albumin was constructed by combining logistic regression. The overall performance of the model was verified to be stable, with good discrimination (AUC = 0.900) and calibration (Hosmer-Lemeshow χ2 = 5.231, *p* = 0.733 > 0.05). As a result, the model can be used as an early warning model for the risk of severe ICH-VTE. In this study, we advocate for the utilization of timely bedside ultrasound by physicians qualified in critical care ultrasound to identify SEC for the purpose of dynamic assessment of ICH-VTE risk and the implementation of stratified management and individualized anticoagulation strategies, with the objective of reducing the incidence of VTE and improving the clinical outcome of critical care ICH.

It is important to acknowledge that the study still presents some shortcomings. The single-center study resulted in a small sample size for inclusion, and the predictive performance of the model requires further validation. Additionally, the acquisition and analysis techniques of SEC images are somewhat subjective, and ensuring the homogenization and standardization of ultrasound images is crucial for the expansion of a multi-center study. Further research will focus on combining SEC with artificial intelligence. This will involve the use of a Multi-Sequence Attention Convolutional Neural Network (MSA-CNN) to automatically acquire, process and analyze lower limb vascular ultrasound images. The aim is to construct artificial intelligence models for critical care ICH-VTE clinical diagnosis and anticoagulation treatment decision-making.

### Limitations

This study lacks internal validation via bootstrapping, which may lead to overestimation of the model’s performance. The limitation stems from the combination of our prospective design’s sample size constraints and the urgent clinical need to report preliminary findings for severe ICH patients, where no validated scores currently exist.

## Conclusion

The severe ICH-VTE risk early warning model, constructed based on SEC, Albumin and age, has been demonstrated to perform stably with good differentiation and calibration. This enables clinicians to assess the risk of severe ICH-VTE dynamically, thus facilitating the provision of stratified management and individualized anticoagulation therapy. Furthermore, the model may provide insights into potential therapeutic strategies for reducing the incidence of VTE and improving the clinical outcome of ICH.

## Data Availability

The original contributions presented in the study are included in the article/Supplementary material, further inquiries can be directed to the corresponding author.

## References

[1] Becattini C Cimini LA Carrier M. Challenging anticoagulation cases: a case of pulmonary embolism shortly after spontaneous brain bleeding. Thromb Res. (2021) 200:41–7. doi: 10.1016/j.thromres.2021.01.016, PMID: 33529872

[2] Zhang R Sun W Xing Y Wang Y Li Z Liu L. Implementation of early prophylaxis for deep-vein thrombosis in intracerebral hemorrhage patients: an observational study from the Chinese stroke center Alliance. Thromb J. (2024) 22:22. doi: 10.1186/s12959-024-00592-w, PMID: 38419108 PMC10900581

[3] Ji R Wang L Liu X Liu Y Wang D Wang W. A novel risk score to predict deep vein thrombosis after spontaneous intracerebral hemorrhage. Front Neurol. (2022) 13:930500. doi: 10.3389/fneur.2022.930500 36388194 PMC9650187

[4] Wang B Wang Z Fu G He B Wang H Zhuo W. Left atrial spontaneous echo contrast and ischemic stroke in patients undergoing percutaneous left atrial appendage closure. Front Cardiovasc Med. (2021) 8:723280. doi: 10.3389/fcvm.2021.723280, PMID: 34631825 PMC8495018

[5] Dugar S Duggal A Bassel A Soliman M Moghekar A. Spontaneous echo contrast in venous ultrasound of severe COVID-19 patients. Intensive Care Med. (2020) 46:1637–9. doi: 10.1007/s00134-020-06094-3, PMID: 32462324 PMC7251216

[6] Ito T Suwa M. Left atrial spontaneous echo contrast: relationship with clinical and echocardiographic parameters. Echo Res Pract. (2019) 6:R65–r73. doi: 10.1530/ERP-18-0083, PMID: 30959476 PMC6499934

[7] Ding D Sekar P Moomaw CJ Comeau ME James ML Testai F. Venous thromboembolism in patients with spontaneous intracerebral hemorrhage: a multicenter study. Neurosurgery. (2019) 84:E304–e10. doi: 10.1093/neuros/nyy333, PMID: 30011018 PMC12311975

[8] Hemphill JC 3RD Greenberg SM Anderson CS Becker K Bendok BR Cushman M. Guidelines for the management of spontaneous intracerebral hemorrhage: a guideline for healthcare professionals from the American Heart Association/American Stroke Association. Stroke. (2015) 46:2032–60. doi: 10.1161/STR.0000000000000069, PMID: 26022637

[9] Mendel R Abdelhameed N Salman RA Salman RA-S Cohen H Dowlatshahi D. Prevention of venous thromboembolism in acute spontaneous intracerebral haemorrhage: a survey of opinion. J Neurol Sci. (2023) 454:120855. doi: 10.1016/j.jns.2023.120855, PMID: 38236754

[10] Barbar S Noventa F Rossetto V Ferrari A Brandolin B Perlati M. A risk assessment model for the identification of hospitalized medical patients at risk for venous thromboembolism: the Padua prediction score. J Thromb Haemost. (2010) 8:2450–7. doi: 10.1111/j.1538-7836.2010.04044.x, PMID: 20738765

[11] Rinaldo L Brown DA Bhargav AG Rusheen AE Naylor RM Gilder HE. Venous thromboembolic events in patients undergoing craniotomy for tumor resection: incidence, predictors, and review of literature. J Neurosurg. (2019) 132:10–21. doi: 10.3171/2018.7.JNS181175 30611138 PMC6609511

[12] Dong C Li Y Ma Z. Venous thromboembolism after spontaneous intracerebral hemorrhage and the status quo of anticoagulation in this population: a retrospective casecontrol study from a tertiary hospital in China. Clin Neurol Neurosurg. (2023) 231:107839. doi: 10.1016/j.clineuro.2023.107839, PMID: 37348314

[13] Fu H Hou D Xu R You Q Li H Yang Q. Risk prediction models for deep venous thrombosis in patients with acute stroke: a systematic review and meta-analysis. Int J Nurs Stud. (2024) 149:104623. doi: 10.1016/j.ijnurstu.2023.104623, PMID: 37944356

[14] Pan X Wang Z Chen Q Xu L Fang Q. Development and validation of a nomogram for lower extremity deep venous thrombosis in patients after acute stroke. J Stroke Cerebrovasc Dis. (2021) 30:105683. doi: 10.1016/j.jstrokecerebrovasdis.2021.105683, PMID: 33676327

[15] Pan X Wang Z Fang Q Li T Xu L Deng S. A nomogram based on easily obtainable parameters for distal deep venous thrombosis in patients after acute stroke. Clin Neurol Neurosurg. (2021) 205:106638. doi: 10.1016/j.clineuro.2021.106638, PMID: 33930795

[16] Liu Z Mi J. Serum albuminumin and circulating metabolites and risk of venous thromboembolism: a two-sample mendelian randomization study. Front Nutr. (2021) 8:712600. doi: 10.3389/fnut.2021.712600 34859025 PMC8631825

[17] Zwart SR Auñón-Chancellor SM Heer M Melin MM Smith SM. Albumin, oral contraceptives, and venous thromboembolism risk in astronauts. J Appl Physiol. (2022) 132:1232–9. doi: 10.1152/japplphysiol.00024.2022, PMID: 35389755 PMC9126217

[18] Arques S. Human serum Albuminumin in cardiovascular diseases. Eur J Intern Med. (2018) 52:8–12. doi: 10.1016/j.ejim.2018.04.014, PMID: 29680174

[19] Linder M Voigtländer L Schneeberger Y Bhadra OD Grundmann D Demal T. Spontaneous echo contrast, left atrial appendage thrombus and stroke in patients undergoing transcatheter aortic valve implantation. EuroIntervention. (2021) 16:1114–22. doi: 10.4244/EIJ-D-20-00743, PMID: 32863242 PMC9725018

[20] Wang H Xi S Chen J Zhao L Gan T He B. Severe left atrial spontaneous echo contrast in nonvalvular atrial fibrillation: clinical characteristics and impact on ischemic risk postablation. Thromb Haemost. (2023) 123:522–34. doi: 10.1055/a-1983-0516 36402133

[21] Huang O Shi Z Garg N Jensen C Palmeri ML. Automated spontaneous echo contrast detection using a multisequence attention convolutional neural network. Ultrasound Med Biol. (2024) 50:788–96. doi: 10.1016/j.ultrasmedbio.2024.01.016, PMID: 38461036 PMC11060922

