# Supplementary material for: Construction of an early warning model for venous thromboembolism risk in patients with severe cerebral hemorrhage based on ultrasound spontaneous imaging
Source: Front Neurol. 2025 Jun 4;16:1562963. doi: 10.3389/fneur.2025.1562963 (PMC12175772; doi:10.3389/fneur.2025.1562963)
Supplement: Supplementary file 1 [file Supplementary_file_1.docx]

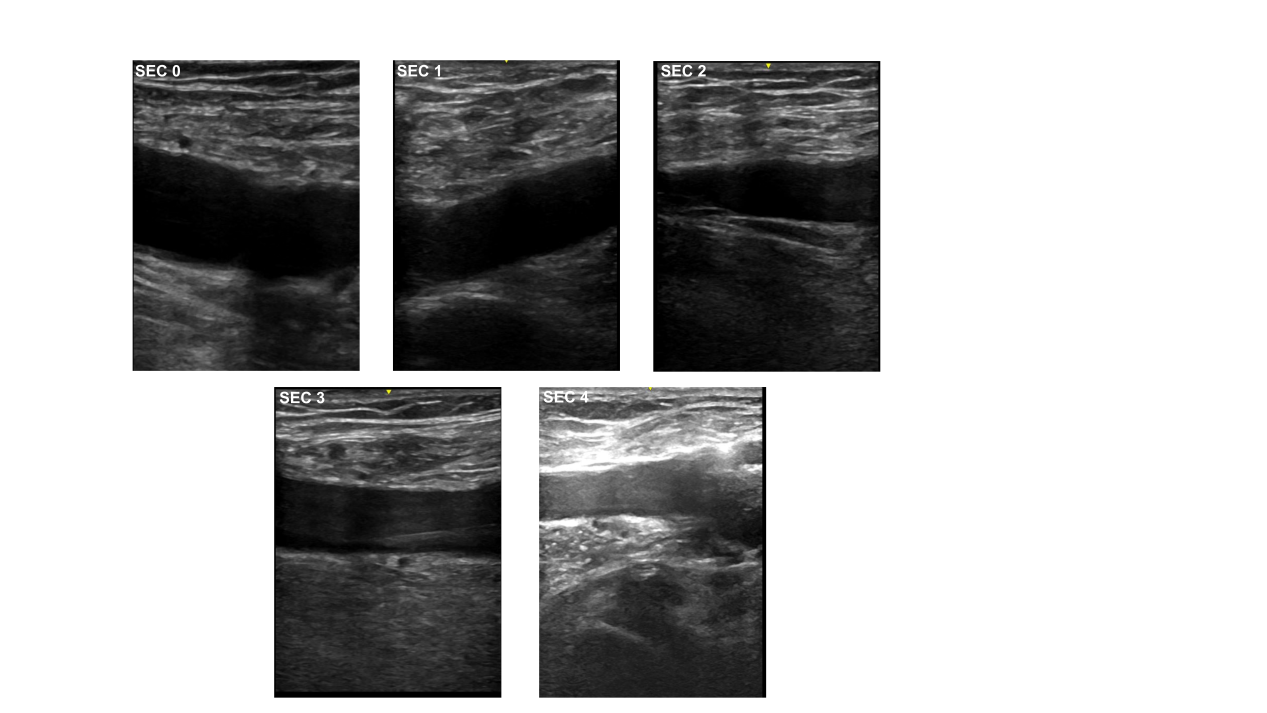


Supplementary figure 1. SEC imaging and grading:Grade 0: Absent;Grade 1: Weak SEC, filling less than 50% of the lumen;Grade 2: Dense SEC, <50% of the lumen;Grade 3: Dense SEC, >50% of the lumen;Grade 4: Dense SEC filling the entire lumen with stagnation

Supplementary table1 .Z-test statistical analysis of multiple ROC curves

| Variables | S.E. | 95%*CI* | *Z* | *P* |
| --- | --- | --- | --- | --- |
| ALB-Age | 0.085 | -0.132 ~0.2048 | 0.421 | 0.673 |
| ALB-SEC | 0.079 | -0.073~0.237 | 1.042 | 0.297 |
| Age-SEC | 0.076 | -0.031~ 0.268 | 1.551 | 0.121 |
| the joint prediction model-ALB | 0.058 | 0.052~0.317 | 2.861 | **0.004** |
| the joint prediction model-Age | 0.067 | 0.071~0.332 | 3.015 | **0.003** |
| the joint prediction model-SEC | 0.034 | 0.017~0.149 | 2.474 | **0.013** |

Supplementary table 2. Comparison of the performance of the Subgroup model

| subgroup | Number | VTE | AUC（95%CI） | Hosmer-Lemeshow P |
| --- | --- | --- | --- | --- |
| Age＜55 | 24 | 4 | 0.988（0.952-1.000） | 0.996 |
| Age≥55 | 45 | 20 | 0.835（0.716-0.955） | 0.413 |
| GCS≤8 | 44 | 16 | 0.891（0.789-0.992） | 0.548 |
| GCS＞8 | 25 | 8 | 0.912（0.795-1.000） | 0.996 |
